# Extended Reality–Enhanced Mental Health Consultation Training: Quantitative Evaluation Study

**DOI:** 10.2196/64619

**Published:** 2025-04-02

**Authors:** Katherine Hiley, Zanib Bi-Mohammad, Luke Taylor, Rebecca Burgess-Dawson, Dominic Patterson, Devon Puttick-Whiteman, Christopher Gay, Janette Hiscoe, Chris Munsch, Sally Richardson, Mark Knowles-Lee, Celia Beecham, Neil Ralph, Arunangsu Chatterjee, Ryan Mathew, Faisal Mushtaq

**Affiliations:** 1 Centre for Immersive Technologies HELIX University of Leeds Leeds United Kingdom; 2 School of Psychology Faculty of Medicine & Health University of Leeds Leeds United Kingdom; 3 School of Science, Technology and Health York St John University York United Kingdom; 4 School of Healthcare Faculty of Medicine & Health University of Leeds Leeds United Kingdom; 5 NHS England England United Kingdom; 6 Fracture Reality Brighton United Kingdom; 7 School of Medicine Faculty of Medicine & Health University of Leeds Leeds United Kingdom; 8 Department of Neurosurgery Leeds Teaching Hospitals NHS Trust Leeds United Kingdom

**Keywords:** mental health, training, consultation, extended reality, virtual reality, augmented reality

## Abstract

**Background:**

The use of extended reality (XR) technologies in health care can potentially address some of the significant resource and time constraints related to delivering training for health care professionals. While substantial progress in realizing this potential has been made across several domains, including surgery, anatomy, and rehabilitation, the implementation of XR in mental health training, where nuanced humanistic interactions are central, has lagged.

**Objective:**

Given the growing societal and health care service need for trained mental health and care workers, coupled with the heterogeneity of exposure during training and the shortage of placement opportunities, we explored the feasibility and utility of a novel XR tool for mental health consultation training. Specifically, we set out to evaluate a training simulation created through collaboration among software developers, clinicians, and learning technologists, in which users interact with a virtual patient, “Stacey,” through a virtual reality or augmented reality head-mounted display. The tool was designed to provide trainee health care professionals with an immersive experience of a consultation with a patient presenting with perinatal mental health symptoms. Users verbally interacted with the patient, and a human instructor selected responses from a repository of prerecorded voice-acted clips.

**Methods:**

In a pilot experiment, we confirmed the face validity and usability of this platform for perinatal and primary care training with subject-matter experts. In our follow-up experiment, we delivered personalized 1-hour training sessions to 123 participants, comprising mental health nursing trainees, general practitioner doctors in training, and students in psychology and medicine. This phase involved a comprehensive evaluation focusing on usability, validity, and both cognitive and affective learning outcomes.

**Results:**

We found significant enhancements in learning metrics across all participant groups. Notably, there was a marked increase in understanding (*P*<.001) and motivation (*P*<.001), coupled with decreased anxiety related to mental health consultations (*P*<.001). There were also significant improvements to considerations toward careers in perinatal mental health (*P*<.001).

**Conclusions:**

Our findings show, for the first time, that XR can be used to provide an effective, standardized, and reproducible tool for trainees to develop their mental health consultation skills. We suggest that XR could provide a solution to overcoming the current resource challenges associated with equipping current and future health care professionals, which are likely to be exacerbated by workforce expansion plans.

## Introduction

As the demand for mental health services in health care systems continues to rise, the need for skilled professionals capable of providing effective mental health consultation and support also increases [1,2]. In the face of changing workforce training requirements (coupled with significant health care workforce expansion plans), there is a growing recognition that the effective implementation of emerging technologies could help overcome some of the logistical and resource-related barriers involved in education and training.

Mental health nursing, in particular, faces distinct challenges that necessitate specialized training solutions. Mental health nurses encounter unique stressors, including high levels of emotional exhaustion, moral distress, and exposure to patient-initiated violence, all of which contribute to job dissatisfaction and high turnover rates [3], which in turn negatively impact workforce stability, patient outcomes, and overall health care service quality [4]. Additionally, mental health nurses often report insufficient opportunities for continuing professional development and limited support from leadership, further compounding retention challenges. Addressing these issues through targeted and innovative training approaches is essential for fostering resilience, enhancing job satisfaction, and improving workforce retention.

Beyond specialist mental health settings, primary care physicians or general practitioners (GPs) also play key roles in managing mental health conditions, with more than a third of general practice consultations involving mental health issues [5]. Effective communication and therapeutic relationships have been shown to significantly influence outcomes, emphasizing the need for better training in interpersonal and empathetic skills for managing mental health conditions in primary care. However, variability in the ability of GPs to detect and manage mental health issues highlights gaps in current training models [6]. As communication forms a central part of mental health treatment, poorly trained clinicians may inadvertently block disclosure of emotional distress, potentially delaying critical interventions [5]. Therefore, innovative training approaches are crucial not only for mental health nurses but also for GPs and other health care professionals involved in mental health consultations.

Traditional training for health professionals in managing mental health problems typically relies on a combination of in-person placements, which employ observation-based learning, and actor-based simulations. While in-person placements provide valuable real-world experience, they often present challenges, such as unpredictable exposure to a diverse range of patient demographics, risks to both students and vulnerable service users, and limited opportunities for structured feedback. Actor-based simulations, on the other hand, are difficult to scale and standardize due to variability in actors’ interpretations of scripts and inconsistencies in their familiarity with specific case studies. These limitations make it challenging to provide health care professionals with the comprehensive training necessary to handle the complexities of mental health consultations. Effective and compassionate mental health consultations require more than procedural knowledge. They demand the ability to empathize, engage in therapeutic communication, and establish a strong patient-provider relationship. To address these needs, training must focus on promoting empathy and compassion while preparing health care professionals to navigate the diverse backgrounds and emotional experiences of patients. However, traditional training methods often struggle to meet these goals due to ethical concerns around exposing students to sensitive cases and the inherent difficulty in replicating the unpredictable dynamics of real-life mental health scenarios.

Advances in a suite of new immersive technologies that go under the banner of extended reality (XR) and include virtual reality (VR) and augmented reality (AR) could be particularly well-suited to address these challenges by providing interactive, standardized, repeatable learning experiences that bridge the gap between theory and practice. VR presents users with a computer-generated environment that immerses them in a fully digitally simulated environment, while AR overlays virtually generated elements onto the real world. The value of XR for health care training has already been demonstrated across various domains, such as surgery [7], physical rehabilitation [8], anatomy [9], and the training of practical skills in nurses [10]. However, the implementation of XR in the training of mental health professionals has lagged.

Given the importance and complexity of training for mental health consultations, coupled with the increasing workload pressure on GPs and mental health nurses to meet the population’s mental health support needs [7], we set out to test whether XR technology could be used to create a training environment to support the development of mental health consultation skills. We reasoned that the ability to deliver standardized repeatable experiences of varied patient encounters (including more rare presentations) in a safe and controlled environment could provide a learning experience that nurtures confidence and competence in consultation skills that augment traditional training.

To assess the potential efficacy of XR in mental health consultation training, we focused on perinatal mental health training, a subspecialty supporting women navigating mental health challenges during pregnancy or the initial postpartum year. This is an area of the mental health service with an urgent training need. The recent report of the Royal College of Psychiatrists [8] highlighted a critical need for comprehensive perinatal training programs across both specialized and general health care services. There is also a notable lack of confidence among perinatal mental health nurses in their capacity to deliver care to women with perinatal mental health challenges, with only a quarter feeling well-equipped to support these women [9]. Trainees also have relatively limited opportunities to train, with a shortage of placement opportunities. A recent review of perinatal mental health education across 32 UK medical schools [10] found that perinatal mental health was not considered a core curriculum topic. Instead, it was typically incorporated as a subtopic within broader topic areas, such as lectures on depression. Given the shortage of staff and limited placements in perinatal mental health, a new training tool that could support the development of the next generation of health care staff could have an immediate impact.

Here, we report on the validation and evaluation of a novel XR training tool developed through a collaboration between software developers and health care staff, including nurses specializing in perinatal mental health and GPs. The simulation presents an interactive virtual patient (“Stacey”) with severe perinatal mental health problems. Stacey is a mother of 2 children, with her youngest child only 4 weeks old, and has a record of mild postnatal depression following her first birth. Low mood, suicidal ideation, and episodes of psychosis add complex layers to her clinical presentation. Users interact with Stacey verbally, and her responses are selected by a human instructor from a range of prerecorded voice-acted clips in an audio repository. We explore the utility of this tool for supporting social and emotional interactions with the simulation, investigate the ease of use for trainers and trainees, and evaluate the impact on cognitive and affective learning.

## Methods

### Overall Approach

We undertook a 2-stage evaluation process that included a pilot study exploring feasibility and a subsequent evaluation of the perinatal mental health XR training experience in terms of learning outcomes and perceptions. In this section, we introduce the simulation platform and training experience and subsequently detail the methods and procedures common to and distinct for each phase of the experiment. It should be noted that the authors involved in developing the content played no role in the evaluation. The analysis was carried out independently by the authors KH, LT, and FM.

This study was not designed as a head-to-head comparison with traditional training approaches. Some participants, particularly those in mental health nursing, had previously received standard training methods (eg, classroom-based teaching, in-person clinical placements, or actor-based role plays), but these forms of training were not systematically assessed here. Instead, the primary aim was to evaluate the feasibility and potential impact of XR as a supplementary training tool. We have thus included the details of traditional training experiences for context but did not incorporate a direct comparative arm in this work.

### XR Simulation

The simulation was built on a platform (“JoinXR”) created by the software developer Fracture Reality. The JoinXR platform was designed to enable multi-user simulation training environments over a range of head-mounted VR or AR displays. In the evaluation, we used the Meta Quest 2 headset (Meta Platforms, Inc) for the VR version of the platform and Microsoft HoloLens 2 (Microsoft Corp) for the AR version.

The human-computer interface within the JoinXR platform was central to facilitating immersive lifelike interactions with the virtual patient. The interface allowed learners to engage through natural voice-based dialogue, processed in real time using instructor-guided responses. The integration of audio feedback, gesture recognition (HoloLens 2), and hand controllers (Meta Quest 2) enabled users to navigate the virtual space intuitively. Learners interacted directly with Stacey, the patient avatar, whose responses, including eye contact, subtle emotional cues, and body movements, were programmed to mimic real-world patient behavior, creating a realistic and contextually relevant learning experience. Users engaged with the simulation through natural voice-based dialogue and through VR controllers (Meta Quest 2) or hand-tracking gestures (HoloLens 2). Nonverbal communication, such as the avatar’s facial expression, body language, and spatial audio, enhanced the simulation’s realism.

**Figure 1:**
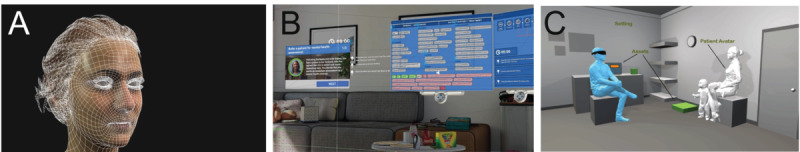
Development of the learning platform. (A) Wireframe of the patient avatar, Stacey; (B) Soundboard for instructors to control Stacey’s responses; (C) Setting for the consultation, showing the learner (blue) and the patient avatar.

The JoinXR platform was designed to be a conversational engine enabling “human to digital avatar” interactions in a multi-user, real-time environment. In this way, it could facilitate remote participation by learners, instructors, and observers, supporting the practice and refinement of nonroutine clinical skills. The learner-instructor dynamic was a crucial component of the simulation, incorporating both real-time guidance and postsimulation feedback. Instructors played an active role during the interaction by interpreting learner inputs and controlling Stacey’s responses using a soundboard system (Figure 1B). This allowed for dynamic adaptations, where learners could engage organically with the avatar and explore different conversational pathways. After the simulation, instructors conducted debrief sessions using performance analytics that tracked response accuracy, emotional sensitivity, and decision-making, providing learners with targeted feedback to refine their clinical competencies.

**Figure 2:**
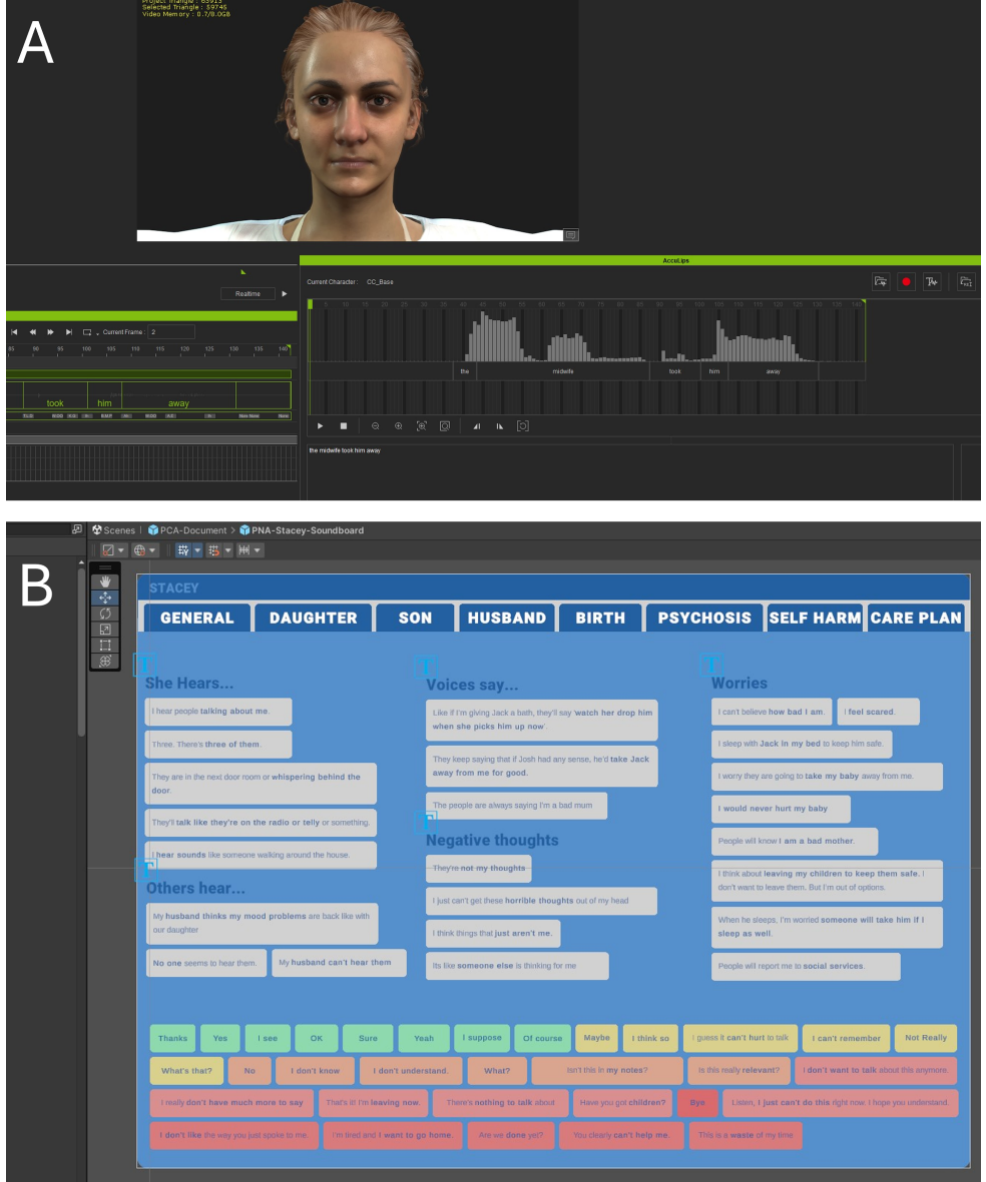
Development of the verbal storyboard and voice integration for patient avatar interactions. (A) Demonstration of the process of integrating voice actors’ performances into the patient avatar through a custom Unity-based lip-syncing system. Multiple iterations of dialogue scripts were recorded, and voice actors were selected via auditions, with audio engineering applied to simulate realistic patient speech patterns. Motion capture, hand animation, and spatial audio balancing enhanced the avatar’s authenticity. (B) Demonstration of the verbal storyboard for the virtual patient, displaying categorized responses covering key clinical themes such as psychosis, self-harm, and family concerns. The storyboard guided the avatar’s realistic conversational flow, informed by clinical expertise and reference material from National Health Service facilities to create immersive training scenarios.

During the simulation, learners interacted with Stacey by asking questions that were either processed by conversational artificial intelligence (AI) or directly controlled by the instructor for tailored responses. Stacey’s reactions were designed to simulate real-world patient behaviors, including nuanced emotional expressions and gestures. Figure 2B illustrates an example consultation scenario in which the learner uses voice input to ask about Stacey’s symptoms, prompting verbal and nonverbal responses (eg, maintaining eye contact and gesturing to emphasize a point).

Learners using VR devices (Meta Quest 2) could navigate the virtual consultation room using handheld controllers to manipulate objects, such as a clipboard or a stethoscope, or to adjust their position relative to Stacey. In contrast, AR users (HoloLens 2) experienced a blended environment where Stacey’s avatar appeared within a real-world room.

The clinical simulations were developed through collaboration between Fracture Reality and a panel of subject-matter experts from the National Health Service (NHS), including mental health clinicians, GPs, and psychologists specializing in perinatal mental health. These experts supported the design of all aspects of the simulations, from character development and storyline construction to ensuring the accurate portrayal of medical conditions. Prior to the present evaluation, the development process included an iterative feedback process involving clinicians with primary care and perinatal mental health experience, software developers, and intended end users.

The specific focus of our evaluation is the first clinical simulation scenario developed using this new platform (Figure 1). The simulation is centered around a female patient avatar (“Stacey”). The aforementioned clinical experts contributed to the development of her patient history and personal attributes. Digital reference photos were then gathered to build a montage of the patient. A base model was built by taking a full body scan of a human model and was modified using a combination of 3D modeling software. The 3D models were created using a combination of Maya (Autodesk) and Blender (Blender Foundation). Clothing was designed, and then, the digital model was dressed. Bespoke custom lighting and skin rendering pipelines were developed to deliver realistic digital human features that could be rendered on headsets using low-powered graphics processing units.

Multiple script iterations were recorded, and the dialogue was reviewed and refined for clinical authenticity by the Fracture Reality team in consultation with the aforementioned subject-matter experts. Auditions were held to select actors. Studio sessions and spatial audio engineering rebalanced vocals to realistically imitate the patient avatar. Animations combined motion capture, hand animation, and lip-syncing for seamless responses. A custom Unity system facilitated quick and accurate lip-syncing to facial expressions and body poses (Figure 2). Reference photos from NHS facilities were used, and lighting was tailored for realistic environments, focusing on meaningful prop placement.

Two scenarios were designed, each tailored to address the needs of 2 primary but distinct target groups: mental health nursing students and primary care trainees (postgraduate doctor in GP training). While both scenarios feature a patient named Stacey presenting with a similar mental health condition, contextual variations were introduced to align more closely with the necessary professional capabilities of the respective trainee groups.

In the mental health nursing scenario, Stacey Morris is introduced as an emergency referral from her GP for a comprehensive assessment. Stacey, a 32-year-old mother of 2 children, with a 4-week-old newborn, has a history of postpartum depression following the birth of her first child. The primary objective for the student in this scenario is to conduct an initial mental health examination of Stacey.

In the primary care scenario, following a telephone conversation with her husband, Josh, who expressed concerns about her behavior, the postgraduate doctor in GP training agrees to meet Stacey in her home. Stacey in this scenario has a similar profile as in the mental health nursing scenario. She is a 32-year-old mother of 2 children, with her youngest child being 4 weeks old. In this context, the role of the postgraduate doctor in GP training centers on conducting a comprehensive mental health assessment with Stacey.

For each context, specific learning outcomes were defined by subject-matter experts. For the mental health nursing scenario, learners were expected to (1) understand and reflect on the lived experience of assessing the mental health of a patient with perinatal mental health problems; (2) identify signs and symptoms of perinatal mental ill health in acute assessment presentation; (3) apply the skills, knowledge, and abilities relevant to one’s own profession in the assessment of mental health; and (4) have an appropriate reflected and evaluated performance of the task in a supported reflection. For the primary care setting, learners were expected to be able to (1) take history from a patient presenting with an acute psychotic illness; (2) ascertain and evaluate information relating to safeguarding; and (3) assess suicide and homicide risk.

### General Methods

Following study advertisement, interested participants were screened for physical conditions that would exclude them from participation, including physical and auditory impairments and epilepsy. Included participants met with the instructor for a one-to-one session in a quiet room located on the university campus or at a local NHS hospital. Multimedia Appendix 1 outlines the study procedure. At the beginning of the session, participants had the opportunity to read the information sheet and ask questions related to the study. Participants provided their consent to the study once they had been informed of their right to withdraw.

After consenting, participants were asked to complete a baseline questionnaire, capturing demographic and attitudinal data. Participants were then randomly allocated to 1 of 2 immersive environments: VR (Meta Quest 2) or mixed reality (HoloLens 2). Participants were subsequently exposed to either “primary care” Stacey (targeted at medical students and postgraduate doctors in GP training) or “mental health” Stacey (for mental health nursing or psychology students), contingent upon their current training program. These simulations share identical features, with the sole distinction lying in the introductory context of the consultation process. Both simulations were configured to align with the familiar protocols of health care trainees, specifically in terms of patient reception. Importantly, the responses of Stacey and the trajectory of the consultation remained consistent across the 2 scenarios.

Trainees verbally interacted with Stacey, who was in turn controlled by an instructor through the navigation of a soundboard, which triggered prerecorded audio clips from Stacey (see Figure 1B). The conversation journey would typically begin with general introductions; discussion of Stacey’s relationships with her daughter, son, and husband; discussion of her birthday; discussion of experiences of psychosis and self-harm; and finally, formulation of a care plan. If the instructor felt the student was unable to lead the conversation or the student expressed having difficulty conversing with the avatar, prompts could be provided within the simulation. Multimedia Appendix 2 shows the prompts available for the early, mid, and late stages of the conversation that could be made visible to the students by the instructor.

Following the experience, the instructor carried out a postexperience debrief session with the trainee, including a critical discussion of the experience and the participant’s performance. Following this, participants completed a postexperience survey measuring attitudinal domains and career considerations alongside measures of usability, presence, discomfort, and preference. The total session lasted approximately 1 hour (Multimedia Appendix 1).

### Pilot Study

#### Participants

In the pilot study, we recruited 9 subject-matter experts from primary care and mental health disciplines. This included a consultant perinatal psychiatrist, a GP, 4 mental health nurses, a specialist perinatal mental health nurse, a psychiatry trainee (ST4), and a mental health nursing lecturer. All had more than 5 years of experience in their respective fields, with 8 having 10 or more years of experience. The purpose of this study was to formally test face and content validity and usability, and to support the latter, we included 5 undergraduate university students (mean age 22.4 years, SD 0.8 years).

Participants followed the procedure outlined in Multimedia Appendix 1. We evaluated face and content validity, usability, and utility as reported by a group of nonnursing or medical students and subject matter experts in the postexperience questionnaire. Face validity was assessed using a scale applied previously to expert evaluations of VR health care training [11]. This original 13-item scale was adapted to this study, and 11 items analyzed the ease of use, effectiveness, and immersion of the XR simulation on a 4-point Likert scale (strongly agree to strongly disagree).

In addition, the Lawshe method [12] also known as the content validity ratio (CVR) method was used. This is a method used to assess the content validity of a measurement instrument or a test, especially in the context of psychological, educational, or health care research, using expert opinion. Here, experts rate each item on a 3-point scale: (1) Essential: if the item is crucial and necessary for measuring the construct; (2) Useful but not essential: if the item is relevant but not critical for measuring the construct; and (3) Not necessary: if the item is irrelevant or not needed for measuring the construct. The CVR is calculated using the equation:



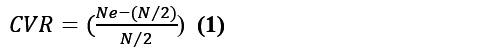



where *Ne* represents the count of experts who have deemed the item as “essential,” and *N* denotes the total number of experts who have participated in the rating process. The CVR is a numerical value that quantifies the consensus among experts regarding the essential nature of the items under consideration. The critical value is a benchmark used to assess the appropriateness of items included in a content validity assessment. If the number of experts who agree on the relevance of an item meets or exceeds the critical value, the item is deemed valid; otherwise, it may be considered for revision or removal from the assessment. According to the values calculated previously [13] with a panel of 8 subject matter experts, this study’s critical value was 0.75. Thus, constructs must surpass a CVR of 0.75 to be deemed essential to the procedure.

We also captured usability through the 10-item System Usability Scale (SUS) [14] as it has widely been used to evaluate XR as a tool for health care training [15-17]. Scores of more than 80 indicate excellence, between 70 and 80 are considered good, and less than 50 are not acceptable [18].

We assessed user discomfort through the Virtual Reality Sickness Questionnaire (VRSQ) [19]. As a more context appropriate adaptation of the validated Simulator Sickness Questionnaire (SSQ) [20], the VRSQ was designed to minimize burden on participants. The VRSQ sums the scores of oculomotor and disorientation discomfort items to generate an overall total. While there are no widely agreed bounds of acceptability for the VRSQ, we set out to compare the scores of the Meta Quest 2 and HoloLens 2 to assess relative differences in physical discomfort between the 2 devices.

### Experiment

Following the demonstration of the feasibility of the use of XR in consultation training, we undertook a larger-scale evaluation. Here, we continued to collect measures of usability and supplemented them with surveys exploring cognitive and affective learning, training preference, presence, and career considerations.

#### Participants

The experiment involved 123 participants (mean age 24.3 years, SD 7.86 years; 97 female participants, 22 male participants, 3 nonbinary or third gender participants, and 1 who did not disclose gender). No participants had known health conditions, such as epilepsy, or visual, auditory, or cognitive disorders that would prevent participation in XR-based activities. They were drawn from a range of health care disciplines, including postgraduate doctors in GP training (n=18; mean age 38.2 years, SD 6.38 years), mental health nursing students (n=30; mean age 25.9 years, SD 7.6 years) from the Universities of Leeds and Huddersfield, and undergraduate medical students (n=28; mean age 19.8 years, SD 3.12 years) and psychology students (n=47; mean age 19.8 years, SD 3.2 years) recruited from the University of Leeds.

Participants were approached via their institutions, through the distribution of emails including information sheets. Participants were offered a monetary voucher incentive where appropriate (ie, for registered students), while clinical staff were asked to undertake the study voluntarily with no remuneration. Participants were randomly allocated to the AR (n=63, 51.2%) and VR training groups (n=60, 48.8%).

#### Measures

In phase 2, the VRSQ and usability continued to be evaluated as described in the pilot study. The experiment extended the evaluation to capture attitudes, cognitive and affective learning, career aspirations, presence, and conversation fluency. These self-reported measures were implemented to provide insights into the user’s social and emotional interactions with the simulation, as well as any reported enhancements in knowledge, understanding, motivation, learning satisfaction, and learning confidence, further assessing the effectiveness of XR mental health consultations.

#### Attitudes

#### Cognitive Learning

Success and confidence in practical situations are often predicted by possessing knowledge, familiarity, and understanding of the themes and techniques embedded in a course curriculum [21]. Conversely, a deficiency in such familiarity may hinder the ability to apply theoretical knowledge in practice [22].

To capture this, a 14-item Perinatal Mental Health Familiarity and Awareness Scale (PMHAFS) (Multimedia Appendix 3) was developed by the study team with subject-matter experts. Participants were asked to evaluate their knowledge with, awareness of, and understanding of the perinatal mental health assessment conditions and care on a 5-point Likert scale (strongly disagree to strongly agree).

#### Affective Learning

Intrinsic and extrinsic motivation for learning was assessed through a 6-item scale [23], developed based on the Motivated Strategies for Learning Questionnaire Manual [24]. Evaluating intrinsic and extrinsic constructs provides a holistic examination of the influences of learner engagement from within the learner and from the learning environment [25]. Higher scores on each item suggest a greater motivation for learning.

To assess self-confidence and learning satisfaction, a 12-item variant [26] of the original Student Satisfaction and Self-Confidence in Learning Scale was used [27]. This instrument has been shown to be highly reliable, with a Cronbach α of .92 for the presence of features and .96 for their importance. Each item on the Likert scale was coded from 1 to 5 (strongly disagree to strongly agree), with 5 items reverse coded to prevent acquiescent responding. Higher scores on the scale indicate greater satisfaction and self-confidence with learning [28].

#### Career Attitudes

To assess students’ considerations of health care specialization, we assessed 9 items across 3 affective domains of motivation, preparedness, and sense of support toward perinatal mental health specialization. Higher scores on each 5-point Likert scale indicate a greater desire to consider perinatal mental health upon graduation.

#### Presence

The construct of presence is regularly evaluated in studies involving virtual environments. Defined as the subjective experience of being in one place or environment, even when physically in another [29], there has been an active debate on its contribution to learning [30-32]. High levels of presence are speculated to be associated with deeper cognitive engagement, a cornerstone for effective learning [29], increasing intrinsic motivation and creating an environment where learners are more likely to integrate and retain new information [32]. A high degree of presence may help to minimize the impact of real-world distractions, allowing learners to fully immerse themselves in the task at hand [33]. Presence has also been proposed to be instrumental for the transfer of skills from the virtual to the real world [31]. We sought to measure presence through the previously validated iGroup Presence Questionnaire (IPQ) [34,35], a 14-item scale capturing spatial presence, realness, and involvement.

### Ethical Considerations

Approval for the study was granted by the School of Psychology Ethics Committee (approval number: PSYC-615; date of approval: November 13, 2022). Consent was obtained from participants at the beginning of the session.

### Statistical Analysis

ANOVA tests were performed to examine the effect of the XR training tool on the ratings of improvement in cognitive learning of conditions, assessment, and care. This same technique was applied to attitude changes in career motivation, support and preparedness, learning confidence, and learning satisfaction. Where appropriate, a between-subjects variable was introduced in the ANOVA when comparing population groups: GP postgraduate doctor in training, mental health nursing student, psychology student, or medical student.

For presence, specific data items related to presence were filtered and selected to include measures, such as “general,” “spatial,” “involvement,” and “realism,” as defined in the IPQ. The presence scores were reported across different devices and groups, examining how users experienced each of these presence measures. An ANOVA assessed differences in presence scores between devices and measures (device [AR vs VR] × iGroup construct [general vs spatial vs involvement vs realism]). Post-hoc tests were applied to decompose interaction effects for VR and AR where appropriate.

For each family of tests (per construct), *P* values were corrected for multiple comparisons using the Bonferroni method. Corrected *P* values below an *α* threshold of .05 were considered statistically significant. All data analyses were performed in R (version 4.2.2) using RStudio (version 2022.12.0.353; Posit).

## Results

### Pilot Study

All experts, across both VR and AR systems (n=9, 100%), felt actively involved and in charge of the situation. The simulation software responded adequately and did not lag according to 8 of the experts, while all 9 experts reported that it was easy to learn how to interact with the software. Notably, all were interested in the progress of events throughout the simulation, suggesting high engagement. Additionally, all stated that it was easy to move around in the virtual environment, and the same amount of people reported that the controller buttons responded adequately.

Using the Lawshe method, we calculated the CVR for each step of the simulation process. These steps were: briefing instructions, medical notes, in-simulation prompts, instructor prompts, and postsimulation debrief. Briefing instructions provided the user with the necessary context for the forthcoming consultation. Medical notes, collaboratively developed with subject-matter experts, provided a comprehensive medical history for the virtual character, Stacey, to enhance the contextual richness of the consultation. In-simulation text prompts, illustrated in Multimedia Appendix 2, could be administered within the XR environment by the instructor, without verbal disruption to the ongoing consultation. In contrast, instructor prompts denoted verbal interventions made by the instructor at any time during the simulation. The postsimulation debrief is an opportunity for the user to reflect and for both the user and instructor to critically evaluate the consultation. The critical value in our study for the content validity of a construct and the component part of the simulation was 0.75. The obtained CVR scores for simulation outcomes, briefing instructions, and postexperience debrief were all 1, indicating that these processes were all rated as essential by all experts.

Some parts of the procedure, including previewing medical notes and using prompts during sessions, were considered optional by design. Our evaluation revealed that all content experts rated it as either essential or useful. In the case of in-simulation and instructor prompts, the majority found them essential or useful, but some considered them “not necessary,” as indicated by a score of 0.75.

The SUS scores were 78.75 for VR and 73.75 for AR, indicating good usability for both systems. The VRSQ scores were 0 for VR and 4.17 for AR, suggesting a negligible amount of discomfort for participants.

### Pilot Study Summary

Participants provided positive feedback, reporting high usability levels for both VR and AR systems and minimal discomfort. Subject-matter experts rated the XR simulation highly in terms of engagement, involvement, and simulation quality, particularly within the context of perinatal training. They found the content and procedures valid, aligning with their expectations for an effective training session. These results suggest that the XR simulation has the potential to serve as a learner-centered training tool and provide the basis for conducting a larger-scale evaluation with health care trainees.

### Experiment

#### Preference

**Figure 3:**
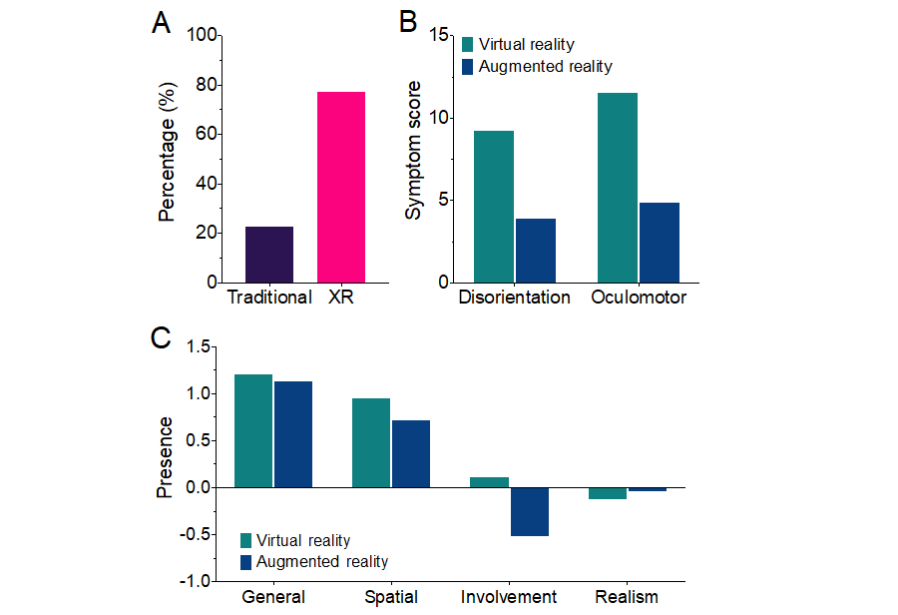
Usability and preference. (A) User preference toward traditional learning for perinatal mental health or the integration of extended reality (XR) to augment perinatal mental health learning. (B) Symptom scores for the disorientation and oculomotor domains of the Virtual Reality Sickness Questionnaire for virtual reality (VR) and augmented reality (AR). (C) Self-reported experience of presence, illustrating that participants felt less involved in AR relative to VR. Error bars represent ±1 SEM.

Participants were asked whether they preferred the XR simulation or traditional approach to training that they had been exposed to. Overall, 77.2% (95/123) of participants preferred XR over traditional training methods (28/123, 22.8%) (Figure 3A).

#### Feasibility

The overall SUS score was 81.6 (SD 11.1), with no difference (*t*_73_=0.75; *P*=.45) between the scores for AR (mean 82.3, SD 10.9) and VR (mean 80.3, SD 12.8), which translates to an excellent usability rating for both systems.

#### Simulator Sickness

In an analysis designed to understand the impact of different devices on simulator sickness, a 2-way ANOVA revealed a significant interaction between device and symptom (*F*_3,312_=6.41; *P*<.001). There were greater sickness scores in the disorientation domain in VR (mean 9.22, SD 1.06) than in AR (mean 3.92, SD 0.81) (*t*_208_=3.47; *P*<.001) and greater scores in the oculomotor domain in VR (mean 11.53, SD 1.33) than in AR (mean 4.89, SD 1.01) (*t*_208_=4.34; *P*<.001). The analysis suggests that VR is more likely to cause symptoms of disorientation and oculomotor discomfort than AR.

#### Presence

IPQ scores were compared between VR and AR. There was a statistically significant interaction between presence measure and device (*F*_3,327_=5.78; *P*=.02; η^2^_G_=0.025). Post-hoc analysis revealed a statistically improved sense of involvement for those using VR relative to AR (*t*_484_=3.18; *P*=.002). There were no significant differences between the systems in general (*t*_484_=0.13; *P*=.90) and in the spatial (*t*_484_=–1.11; *P*=.27) and experienced realism (*t*_484_=1.17; *P*=.24) domains of presence.

#### Learning Outcomes

**Figure 4:**
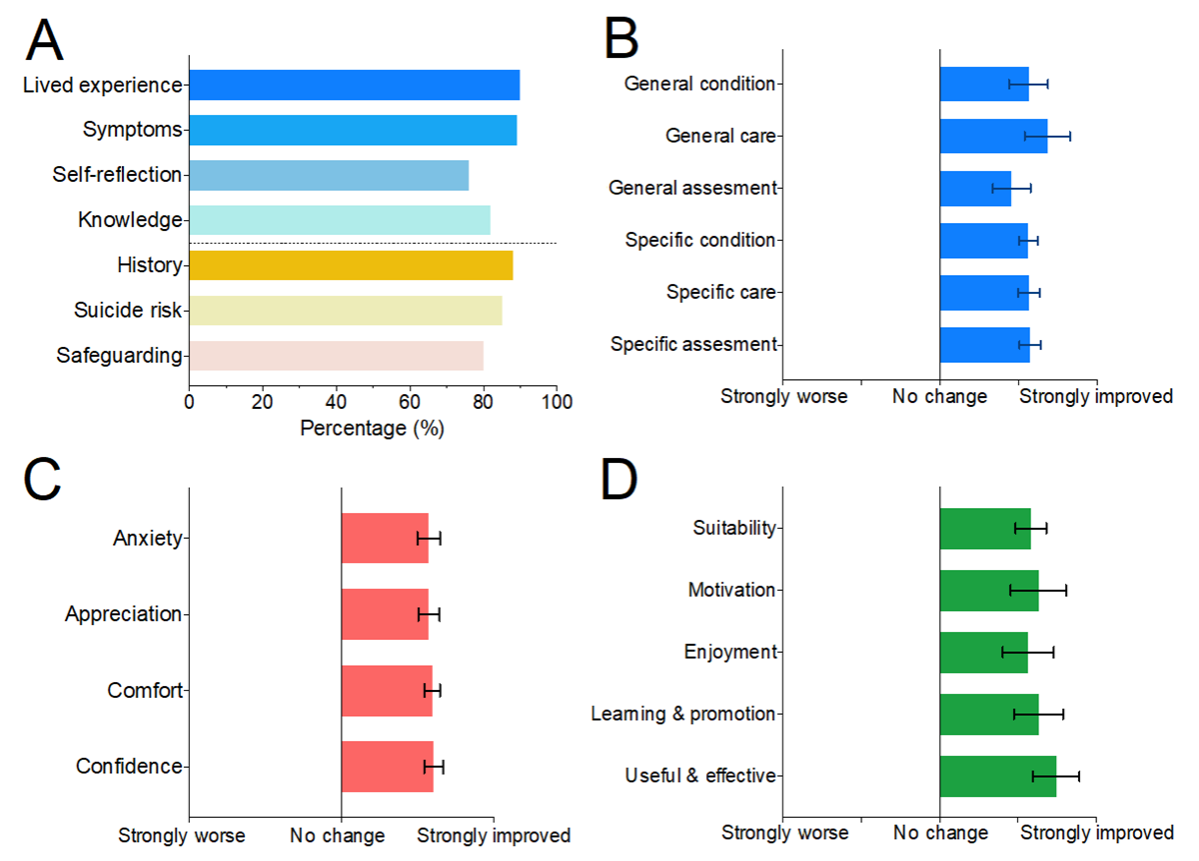
Learning outcomes, cognitive and affective changes, and learning satisfaction. (A) Percentage of achievement of learning objectives for perinatal mental health and primary care simulations within sessions across all participants. (B) Improvements in understanding across perinatal conditions, assessment, and care domains following the simulation for general practitioner (GP) trainees, and improvements in understanding across perinatal conditions, assessment, and care domains following the simulation for mental health nursing students. (C) Improvements in the affective domains of confidence, comfort, appreciation for the challenges in providing support to perinatal cases, and reduction in anxiety among perinatal cases for GP trainees. (D) Improvements in the utility and feasibility domains for mental health consultation training following XR simulations compared with the current training approach. Error bars represent the SEM for domain change responses.

For the 2 simulations evaluated in this study, specific learning outcomes were defined by subject matter experts from perinatal mental health and primary care. We report these separately for each group. Figure 4A shows the percentage of sessions in which the learning outcome was achieved across all groups, as reported by the instructor.

In the primary care simulation, instructors rated that they were able to achieve learning objective 1 (able to take history from a patient presenting with an acute psychotic illness) in 100% of sessions, learning objective 2 (able to ascertain and evaluate information relating to safeguarding) in 80% of sessions, and learning objective 3 (able to assess suicide and homicide risk) in 80% of sessions.

In the perinatal mental health simulation, instructors rated that they were able to achieve learning objective 1 (understand and reflect on the lived experience of assessing the mental health of a patient with perinatal mental health problems) in 100% of sessions, learning objective 2 (identify the signs and symptoms of perinatal mental ill health in acute assessment presentation) in 90% of sessions, learning objective 3 (apply the skills, knowledge, and abilities relevant to one’s own profession in the assessment of mental health) in 89% of sessions, and learning objective 4 (have appropriate reflected and evaluated performance of the task in a supported reflection) in 80% of sessions.

### Changes in Cognitive and Affective Attitudes

#### Primary Care

At baseline, 22% of participants stated that they had “no experience” with perinatal mental health cases, 61% expressed “little experience,” and only 17% expressed “some experience.” Understanding of complex mental health (general) and perinatal mental health (specific) was measured at baseline, revealing that 59% of GP trainees expressed an understanding of complex mental health at a general level and 58% expressed an understanding of perinatal mental health specifically.

Regarding affective constructs, 44% of trainees expressed anxiety around complex mental health cases and 50% expressed anxiety around perinatal mental health cases. Following the simulation, participants reported a statistically significant improvement in cognitive attitudes (mean 0.91, SD 0.86; *t*_17_=4.47; *P*=.003; *d*=1.05).

Participants further reported a statistically significant improvement in affective attitudes following the simulation (mean 0.92, SD 0.74; *t*_17_=5.27; *P*<.001; *d*=1.17). Across the affective domain, participants reported an improvement in confidence (mean 0.83, SD 1.04; *t*_17_=3.39; *P*=.004; *d*=0.79), comfort (mean 0.89, SD 0.76; *t*_17_=4.97; *P*<.001), appreciation for the challenges of providing perinatal mental health support (mean 0.94, SD 1.00; *t*_17_=4.01; *P*=.001; *d*=0.95), and reduced anxiety toward perinatal mental health cases (mean 1.00, SD 1.09; *t*_17_=3.91; *P*=.001; *d*=0.92).

#### Medical Students

Following the simulation, medical students reported an improvement in cognitive attitudes (mean 1.38, SD 0.40; *t*_27_=18.14; *P*<.001; *d*=3.42). This group also reported a statistically significant improvement in affective attitudes (mean 1.35, SD 0.46; *t*_27_=10.01; *P*<.001; *d*=1.89). Across the affective domain, students reported an improvement in confidence (mean 1.64, SD 0.58; *t*_27_=13.45; *P*<.001; *d*=2.54), comfort (mean 1.39, SD 0.57; *t*_27_=13.00; *P*<.001; *d*=2.46), appreciation (mean 1.29, SD 0.90; *t*_27_=7.59; *P*<.001; *d*=1.43), and reduced anxiety toward perinatal mental health cases (mean 1.25, SD 0.97; *t*_27_=6.84; *P*<.001; *d*=1.29).

#### Mental Health Students and Psychology Students

Mental health and psychology students reported a significantly improved understanding of perinatal mental health conditions (mean 1.01, SD 0.62; *t*_76_=14.26; *P*<.001; *d*=1.63), assessment (mean 1.21, SD 0.74; *t*_76_=14.60; *P*<.001; *d*=1.62), and care (mean 1.09, SD 0.65; *t*_26_=14.82; *P*<.001; *d*=1.69) following the simulation.

Within the mental health student group, improvements were seen following the simulation across the domains of perinatal mental health conditions (mean 0.86, SD 0.65; *t*_29_=7.26; *P*<.001; *d*=1.32), assessment (mean 0.91, SD 0.67; *t*_29_=7.41; *P*<.001; *d*=1.35), and care (mean 0.76, SD 0.60; *t*_29_=6.88; *P*<.001; *d*=1.26).

Improvements were also seen in the psychology group across the domains of conditions (mean 1.11, SD 0.59; *t*_46_=12.85; *P*<.001; *d*=1.87), assessment (mean 1.39, SD 0.73; *t*_46_=13.10; *P*<.001; *d*=1.91), and care (mean 1.31, SD 0.59; *t*_26_=15.32; *P*<.001; *d*=2.23).

Across all mental health and psychology students, we found a significant increase in learning confidence (mean 1.14, SD 0.49; *t*_76_=20.32; *P*<.001; *d* =2.32). Students further reported a significant increase in learning satisfaction (mean 1.33, SD 0.69; *t*_76_=16.51; *P*<.001; *d*=1.88). There was a similar finding within groups, as mental health students reported a significant increase in learning confidence following the simulation (mean 1.13, SD 0.57; *t*_29_=10.83; *P*<.001; *d*=1.98). Psychology students also reported a significant increase in learning confidence following the simulation (mean 1.13, SD 0.43; *t*_46_=15.32; *P*<.001; *d*=2.61).

For learning satisfaction, mental health students reported a significant increase following the simulation (mean 1.25, SD 0.82; *t*_29_=8.33; *P*<.001; *d*=1.52), and psychology students also reported a significant increase following the simulation (mean 1.37, SD 0.62; *t*_46_=15.12; *P*<.001; *d*=2.20).

#### Career Considerations

At baseline, 49% of mental health nursing students stated that they were motivated to pursue a career in perinatal mental health, while 30% agreed that they felt prepared to pursue a career in perinatal mental health and 24% felt supported to pursue a career in perinatal mental health. Only 25% of psychology students were considering a career in perinatal mental health. Following the simulation, mental health nursing students felt significantly more motivated (mean 0.73, SD 0.65; *t*_29_=6.15; *P*<.001; *d*=1.12), prepared (mean 1.10, SD 0.52; *t*_29_=11.61; *P*<.001; *d*=2.12), and supported (mean 0.74, SD 0.74; *t*_29_=5.79; *P*<.001; *d*=1.06) to pursue a career in perinatal mental health. Similarly, psychology students also reported a significantly greater likelihood of considering a career in perinatal mental health following the simulation (*t*_46_=7.04; *P*<.001; *d*=1.03).

#### Instructor Training

**Figure 5:**
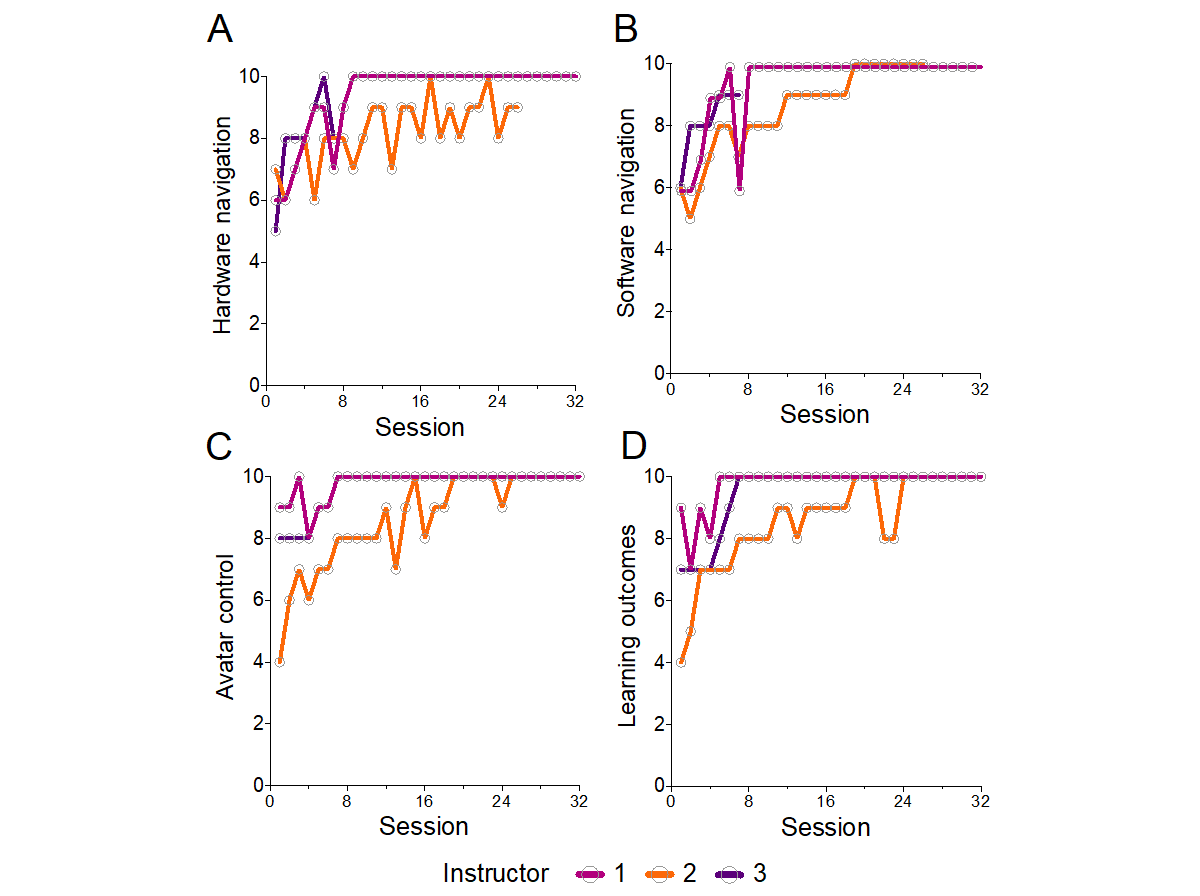
Instructor development over session delivery. Following each session, instructors self-reported the following on a scale of 1 to 10: (A) ease of hardware handling; (B) ease of software navigation; (C) confidence in controlling the avatar; and (D) comfort in achieving learning outcomes.

In addition to assessing the benefits for participants, to better understand how much time it would take to train staff without previous XR experience to become comfortable with navigating through this training platform, we asked our instructors to document their degree of confidence on a scale from 0 to 10 regarding four key dimensions: (1) hardware navigation, (2) software navigation, (3) avatar control, and (4) delivery of session learning outcomes. Following each session, instructors assigned ratings to these constructs, thereby creating a subjective trajectory of their session delivery proficiency (Figure 5). Notably, these ratings rose rapidly and plateaued after approximately 6 to 8 sessions across all key constructs, suggesting that it will take multiple training sessions before instructors feel that they can deliver reliably consistent training sessions. We also observed some variation from session to session, which may be accounted for by a combination of measurement errors and technical and logistical factors. While not amenable to formal statistical analysis, instructors reported lower scores when they experienced Wi-Fi dropouts or software crashes.

## Discussion

### Principal Findings

We explored the idea that XR technologies could support the delivery of mental health training through a simulated mental health consultation, in which a trainee interacts with a human-controlled virtual avatar. An initial feasibility pilot with subject-matter experts and students demonstrated potential efficacy worthy of further investigation. We subsequently followed this up with a comprehensive evaluation of its impact on trainees from across mental health nursing, medical doctors training to be GPs, and undergraduate psychology and medicine students. Our findings demonstrate the significant potential of XR as a pedagogical tool in supporting the development of mental consultation delivery skills.

We observed notable enhancements in cognitive and affective learning across all health care trainee groups. Instructors reported high rates of successful delivery of learning objectives, while participant groups reported increased knowledge in diverse perinatal domains, including the recognition of conditions, such as depression and anxiety, during pregnancy and in the postpartum period. Trainees demonstrated proficiency in systematic evaluation using diagnostic tools to assess severity. We also observed improvements in knowledge and confidence, specific to perinatal mental issues and broader issues of working with complex mental health challenges.

The integration of XR into mental health training represents a significant advancement, offering immersive, interactive, and repeatable learning environments that traditional methods often fail to replicate effectively. Conventional training typically relies on static case studies, peer-based role play, or interactions with real patients, each of which presents limitations. XR, however, combines high-quality instructional content with advanced technological features, including real-time feedback, iterative “fail and retry” opportunities, and high-fidelity simulations. This integration is not merely a shift in delivery medium but a holistic synthesis of content and technology, fostering experiential and contextually relevant learning.

The educational content needed to address complex and sensitive scenarios, such as perinatal mental health, is notably limited within mainstream mental health nursing curricula and GP training. Traditional training often emphasizes general psychiatric principles or common conditions, leaving significant gaps in specialized instructions for nuanced cases like acute postpartum psychosis and perinatal depression. This lack of exposure to high-risk sensitive clinical contexts underscores the need for innovative training solutions that can bridge this gap. XR-based simulations offer a tailored and immersive approach, allowing learners to engage with realistic perinatal mental health cases and gain practical experience-driven insights beyond what conventional programs typically provide.

Although this study did not conduct a direct comparison with conventional approaches, XR’s ability to standardize and replicate complex scenarios addresses many logistical and ethical challenges associated with actor- or patient-based training. By facilitating autonomous practice, enhancing critical competencies, and building learner confidence in a psychologically safe environment, XR provides a valuable environment for high-stake contexts such as mental health consultations. Effective training in this domain is essential, as errors can negatively impact both therapeutic relationships and patient outcomes [36]. XR’s capacity to support the development of these therapeutic relationships is key to achieving improved health outcomes for individuals with mental illness [37]. This work also suggests that immersive educational technologies might be able to influence career planning and specialization. Our study found an increase in the reported interest among trainees considering a career in perinatal mental health. This positive shift in attitude toward perinatal mental health careers is particularly significant given the documented shortage in this specialty [9]. Such tools may extend beyond traditional educational outcomes to influence career aspirations and potentially bridge the gap between abstract career concepts and tangible professional identity formation.

Immersive educational technologies, exemplified by XR simulations, possess the potential to not only shape career preferences but also address significant concerns regarding the cultivation of empathetic connections and the practical application of theoretical knowledge during training. In mental health training, a crucial aspect involves nurturing the user’s ability to establish therapeutic relationships. This necessitates engaging in specific scenarios and subsequent reflection to ensure nurses can comprehensively apply theoretical knowledge effectively [38]. We were concerned that the interaction with a virtual avatar may be a poor substitute for the development of this relationship and that it may be difficult to empathize with. However, our investigation into users’ social and emotional interactions within the simulation revealed positive indicators, including general and spatial presence and improvements across cognitive and affective domains. These promising outcomes suggest that immersive technologies may not act as barriers but instead as facilitators in establishing effective therapeutic relationships.

Further grounds for our concerns about the feasibility of this tool in this context came from the “Uncanny Valley” [39] phenomenon, which describes the sense of unease or discomfort experienced when an artificial representation closely resembles a human but is not quite convincingly lifelike. Stacey had indeed been designed to be as realistic as possible (working within the graphical constraints of today’s technology). Our outcomes indicate that the design quality and the method for interacting with the avatar were sufficient to circumvent this effect, allowing users to transcend potential unease and engage meaningfully with the simulation. Nevertheless, somewhat paradoxically, as the graphical capabilities of XR technology increase, this area will become increasingly more important to monitor in the design and implementation of patient avatars until they become indistinguishable from real humans. This necessitates a careful iterative approach in the design and implementation of patient avatars, one that is cognizant of these psychological effects. Future iterations of XR simulations must be not only technically advanced but also underpinned by a deep understanding of user psychology to ensure that they support rather than detract from the learning objectives [40].

In looking to the future, the rapid advances being made in generative AI provide an avenue for such training tools to become increasingly autonomous, which could significantly alleviate the workload of instructors while simultaneously enhancing the dynamic interactivity of training sessions through the development of bespoke patient avatars tailored to the needs of learners. AI analysis of utterance-response pairs could predict context-specific reactions, enabling intelligent and adaptive XR training tools. XR training tools could leverage this “generative” AI to create dynamic and realistic scenarios for training health care professionals in mental health consultations, thereby enhancing their ability to understand and respond to a wide range of patient interactions. The use of generative AI could also democratize access to high-quality training resources, making them available across different geographies and socioeconomic contexts, thereby potentially reducing disparities in mental health training quality globally. Instructors could personalize scenarios, offer real-time feedback, and adapt to unique learner needs. Such potential advances do, however, raise ethical concerns [41], including the risk of bias that would need to be tackled for effective, efficient, and inclusive training.

### Limitations

It is important to note that this study does not suggest that XR learning can replace traditional placements or direct learning opportunities and experiences or that simulation avatars can fully replicate real patients. What it does show is that XR could be a valuable tool for providing standardized training experiences to mental health trainees across different institutions and professional domains. The simulation employed in this study serves as a potential solution for exposing trainees to complex and nonroutine patient presentations. Going a step further, we suggest that the tool could also offer an opportunity to explore underrepresented scenarios, including those involving minoritized populations, and could be a useful vehicle for promoting cultural competence and enhancing the overall diversity of training scenarios. We propose that by using XR technology, mental health training programs may be able to bridge gaps in exposure to various clinical scenarios and populations, contributing to a more comprehensive and inclusive approach to mental health training.

While this study demonstrated significant improvements in various aspects of trainee confidence and perceived competence, it is important to clarify that the study’s primary aim was not to evaluate current educational provisions or compare XR training directly to traditional methods. Instead, the focus was on assessing the feasibility and potential benefits of an XR-based tool as a supplementary learning aid within existing training frameworks. The intention was to explore how XR could augment current educational experiences rather than to position it as a replacement for established training methods. Future research should consider comparative studies that directly assess the effectiveness of XR against traditional pedagogical approaches to determine the conditions under which XR-based learning is most beneficial. Incorporating controlled trials and longitudinal assessments would further strengthen the understanding of XR’s role in skill retention and clinical application. It is also important to note that our evaluation only involved a single session and an examination of changes immediately after the session. This has shown substantial promise and must be followed up with an examination of any longer-term changes, capturing skill retention and whether this knowledge and confidence can be translated to clinical practice. Equally, the implementation of this technology into the curriculum should not be a “one-shot” standalone affair. Instead, we propose that it should be integrated systematically across multiple sessions to reinforce and build upon the acquired knowledge and skills. Long-term evaluations, including follow-up assessments at intervals beyond the immediate postsession period, are imperative to gauge the durability and sustainability of the observed impacts. Additionally, future research endeavors should explore the application of XR technology in diverse clinical scenarios to assess its versatility and effectiveness across various health care contexts. The iterative and continuous integration of XR simulations into the curriculum, coupled with ongoing assessments, will contribute to a more comprehensive understanding of its benefits and practical applicability in real-world health care settings.

While we focused on evaluating one-to-one sessions, the platform also affords the delivery of one-to-many training sessions and the opportunity for group-led discussion. One-to-one sessions in XR offer personalized interactions where trainees can practice engaging with virtual patients in a safe controlled environment, receiving tailored feedback from instructors. This approach allows for intensive skill development, particularly in handling complex or sensitive mental health scenarios. On the other hand, one-to-many sessions leverage XR’s multi-user capabilities to enable group training, where multiple participants can observe and interact within the same virtual environment. By leveraging its multi-user capabilities, XR training tools could be used to create an environment conducive to collaboration, group discussion, and the promotion of intra- and interprofessional discussions.

Furthermore, in a world where hybrid (or blended) learning has started to become a norm, XR provides a practical solution for overcoming resource and time constraints faced by training programs. The ability to access training sessions and share the same learning space from anywhere in the world could provide a practical solution to the resource and time constraints faced by training programs, promoting both inclusivity and efficiency in health care education. This flexibility ensures that trainees across diverse locations and professional domains can participate in standardized training experiences, contributing to equitable and scalable mental health education.

While immersive technologies present transformative opportunities as learning tools, accessibility for individuals with visual and auditory impairments remains a critical concern. XR environments heavily rely on visual and auditory inputs, which can exclude users with disabilities if not adequately addressed. For visual impairments, accessibility may involve features, such as screen reader compatibility, audio descriptions, and haptic feedback, to convey spatial or contextual information. For auditory impairments, captions, subtitles, and integration with assistive hearing devices, such as cochlear implants, are essential. To address these challenges, the software in this study integrates specific accessibility features. For users with hearing impairments, the JoinXR platform includes automatic captioning, enabling subtitles to appear beneath a user’s avatar during interactions. For users with visual impairments, the design process emphasized hardware compatibility, recommending the HTC Vive Focus 3 for VR due to its adjustable lenses and focal settings and the Microsoft HoloLens 2 for AR, which allows users to keep their glasses on. These measures reflect a commitment to inclusivity, though further advancements are needed to fully overcome accessibility barriers in XR technologies.

Finally, important considerations for the implementation of XR training tools are the economic cost and the return on investment. There are significant start-up expenditures, including the procurement of XR hardware and software licenses. In addition, adopting XR technology requires appropriate technical infrastructure, such as accessible, reliable, and reasonably fast internet connectivity, along with a long-term strategy for sustainable implementation. The rapid pace of technological advancement poses the risk of hardware and software becoming quickly obsolete, compelling organizations to contemplate strategies for regular updates and maintenance to keep pace with technological innovations. On the other hand, XR technology affords numerous opportunities to enhance educational experiences, reducing training time and improving learning efficacy [42]. Additionally, the potential of XR to facilitate remote learning could reduce the necessity for travel and accommodation expenses, which are traditionally associated with centralized training programs [43]. A critical next step for advancing this field is the development of a return-on-investment framework. This framework should account for the wide spectrum of benefits as well as the initial and ongoing expenses. In this way, organizations will have clear insights into the viability and value of adopting tools, such as the one introduced here, as they address the escalating demands of health care workforce training.

### Conclusions

The use of an XR-based simulated mental health consultation scenario, where trainees interacted with a human-controlled virtual avatar, showed promise in an initial feasibility pilot and was further substantiated by a comprehensive evaluation across various health care trainee groups. Our findings indicate significant enhancements in cognitive and affective learning, with high rates of successful delivery of learning objectives. These findings show, for the first time, that XR can be used to provide an effective, standardized, and reproducible tool for trainees to develop their mental health consultation skills. We suggest that XR could provide a solution to overcome the current resource challenges associated with equipping current and future health care professionals, which are likely to be exacerbated by workforce expansion plans.

## Appendix

Multimedia Appendix 1 Process flow chart of the hour-long evaluation session.

Multimedia Appendix 2 In-simulation prompts available to the instructors to support users.

Multimedia Appendix 3 Perinatal Mental Health Familiarity and Awareness Scale (PMHAFS).

## Abbreviations

AI: artificial intelligence
AR: augmented reality
CVR: content validity ratio
GP: general practitioner
IPQ: iGroup Presence Questionnaire
NHS: National Health Service
SUS: System Usability Scale
VR: virtual reality
VRSQ: Virtual Reality Sickness Questionnaire
XR: extended reality
