# Supplementary material for: Extended Reality–Enhanced Mental Health Consultation Training: Quantitative Evaluation Study
Source: JMIR Med Educ. 2025 Apr 2;11:e64619. doi: 10.2196/64619 (PMC12004025; doi:10.2196/64619)
Supplement: Multimedia Appendix 1 [file mededu_v11i1e64619_app1.docx]

**Multimedia Appendix 1.** Process flow chart of the hour-long evaluation session.


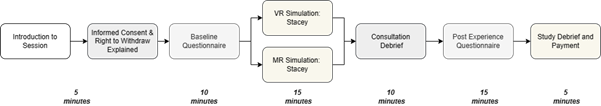


Each segment of the session is delineated with the allocated time displayed beneath. Participants were introduced and provided informed consent, with their right to withdraw explained. Baseline measures were collected through a questionnaire. Subsequently, participants engaged in a simulation, either in Virtual Reality (VR - Oculus Quest 2) or Mixed Reality (AR - HoloLens 2). Following the session, participants participated in a debriefing session with their instructor. The post-experience questionnaire was then administered, preceding a study debrief and, if applicable, participant were remunerated for their participation.
