# Supplementary material for: Extended Reality–Enhanced Mental Health Consultation Training: Quantitative Evaluation Study
Source: JMIR Med Educ. 2025 Apr 2;11:e64619. doi: 10.2196/64619 (PMC12004025; doi:10.2196/64619)
Supplement: Multimedia Appendix 2 [file mededu_v11i1e64619_app2.docx]

**Multimedia Appendix 2.** In-simulation prompts available to the instructors to support users.


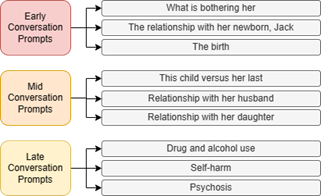


Prompts are organized, within the simulation to support early, mid and late stages of the consultation conversation.
