# Supplementary material for: Extended Reality–Enhanced Mental Health Consultation Training: Quantitative Evaluation Study
Source: JMIR Med Educ. 2025 Apr 2;11:e64619. doi: 10.2196/64619 (PMC12004025; doi:10.2196/64619)
Supplement: Multimedia Appendix 3 [file mededu_v11i1e64619_app3.docx]

**Multimedia Appendix 3.** Perinatal Mental Health Familiarity and Awareness Scale (PMHAFS).

Q: Following the XR experience how do you feel about the following statements? Responses were rated on 5-point scale (Strongly Disagree, Somewhat Disagree, No change, Somewhat agree, Strongly Agree).

1. I am more aware of the conditions that contribute to a perinatal mental health situation
2. I am not more aware of the conditions in determining a perinatal mental health situation
3. I am more familiar with the perinatal mental health clinical environment
4. I am not more familiar the conditions in determining a perinatal mental health situation
5. I have a better understand the conditions surrounding perinatal mental health
6. I do not have a better good understanding of the conditions surrounding perinatal mental health
7. I am more aware of how to assess a clinical perinatal situation
8. I am less familiar with how to assess a clinical PMH situation
9. I have a better understanding of how to assess a perinatal mental health situation
10. I do not have a better understanding of how to assess the perinatal mental situation
11. I am more aware of how to care for a patient in a perinatal mental health situation
12. I am less familiar with how to care for a patient in a perinatal mental health situation
13. I better understand how to care for a patient in a perinatal mental health case
14. I have a better understanding of how to care for a patient in a perinatal situation
